# Building supply chain capacity for neglected tropical diseases: experience from the Ascend West and Central Africa programme

**DOI:** 10.1093/trstmh/trab068

**Published:** 2021-05-15

**Authors:** Rocio Villacorta Linaza, Timothy Garner, Chantelle Genovezos

**Affiliations:** Department of Tropical Disease Biology, Liverpool School of Tropical Medicine, Pembroke Place, L3 5QA, UK; Department of Tropical Disease Biology, Liverpool School of Tropical Medicine, Pembroke Place, L3 5QA, UK; Hypha Group Limited, 320 Firecrest Court Centre Park, Warrington, UK, WA1 1RG, UK

**Keywords:** capacity building, health systems, NTD supply chain, pharmacists, preventative chemotherapy, reverse logistics

## Abstract

The Ascend West and Central Africa programme, funded by the UK Foreign, Commonwealth and Development Office (FCDO) is supporting integrated preventative chemotherapy for up to five neglected tropical diseases (NTDs), including intestinal worms, lymphatic filariasis, river blindness, trachoma and schistosomiasis. The programme is implemented across 13 countries by a consortium of four leading international development partners: Sightsavers, Liverpool School of Tropical Medicine, Schistosomiasis Control Initiative Foundation and Mott Macdonald. This paper presents messages learnt from country assessments that took place prior to the global outbreak of coronavirus disease 2019 (COVID-19). These messages remain relevant post-COVID-19, with greater priority being given to the challenges for national NTD programmes in continuing to deliver mass drug administration (MDA) during the pandemic. Stakeholder coordination from the earliest stages of the pandemic has occurred at two levels: in the first mile with global partners of the NTD Supply Chain Forum and in the last mile with implementing partners in each country. This has been instrumental to manage delayed MDA, including the impact delays have on the shipment of NTD donated drugs and the distribution of stock held in country. The Ascend West and Central Africa programme is supporting countries with the resumption of MDA through a risk assessment and mitigation action (RAMA) process.

## Introduction

Health system strengthening is a key component of the Ascend West and Central Africa programme, which encompasses building supply chain capacity to improve last mile drug availability and data. In this context, the last mile refers to the in-country procedures leading up to the final point of distribution to communities. Overall, Ascend aims to positively impact the sustainability of national neglected tropical disease (NTD) programmes. Building supply chain capacity within ministries of health aims to address the challenges faced by national NTD programmes.

Integrated preventative chemotherapy and transmission control (PCT) is the principal strategy promoted by the World Health Organisation (WHO) for the safe, simple and effective control and elimination of a group of the most prevalent NTDs. Treatment is delivered via mass drug administration (MDA) to entire at-risk populations or to specific at-risk groups, such as school-age children.^1^ Record-breaking commitment for drug donation by pharmaceutical companies to endemic countries and the service delivery model of volunteer community drug distributors (CDDs) enables cost-effective, large-scale PCT programmes to be delivered. Furthermore, the WHO estimate that at least 1.76 billion people still require interventions against NTDs.^2^

To date, more than 1 billion people have received treatment for at least one NTD.^3^ However, increased volume of drug donations alone is neither sustainable nor sufficient to control and eliminate NTDs. How to integrate a comprehensive approach, which combines MDA with on-demand access to treatment, with NTDs packaged into universal health coverage and national health systems, is a challenge that needs further research and action.^4^

To be fully effective, national health systems require capacity at all levels, especially in the last mile of the supply chain. For low- and middle-income countries (LMICs), the infrastructure and distribution networks, political and environmental context, as well as the involvement of many stakeholders and partners, is particularly complex. It is hindered by a lack of human resources, adequate funding and physical infrastructure such as road networks and storage facilities. The risks of not addressing these challenges include stock-outs, wastage, loss and/or theft of tablets, and potentially delayed or missed treatment. Furthermore, effective management of the NTD supply chain directly impacts on the costs and effectiveness of national NTD programmes, supporting elimination targets and value-for-money objectives. Collaboration and investment in the NTD supply chain generates significant benefits, as demonstrated in the first mile though a unique public-private partnership (the NTD Supply Chain Forum). Furthermore, the role of the forum and its resilience have been tested and affirmed most recently during the unprecedented times of the coronavirus disease 2019 (COVID-19) pandemic.^5^

Focus on supply chain capacity and sustainability is consistent with the WHO Road Map for NTDs 2021–2030 and its companion document, the WHO NTD Sustainability Framework for Action (Box 1), which aims to address the gaps in elimination of NTDs. Access, logistics and integration are three of the areas highlighted for crucial action. These are intrinsically linked to health system strengthening through improving the capacity to deliver interventions on the ground.

Box 1. **Supply chain extracts from key global NTD documents**

**WHO Road Map for NTDs 2021–2030** ^2^

• An integrated approach to NTD activities is expected to result in better health outcomes, greater cost efficiency and effectiveness and better programme management

• Integration of the NTD supply chain with national medicine supply networks

• Integrated approaches to NTDs can and should be mainstreamed within various components of national health systems; … delivery of medicines should be coordinated through national medicines supply and logistics systems

• Remaining challenges of improving ‘last-mile’ delivery, integrating provision of NTD medicines and products and improving the transparency of the supply chain

• Need for a strong, responsive supply chain

**WHO NTD Sustainability Framework for Action 2021–2030** ^6^

Sustainability will depend on the continuity of these [PC-NTD drug] donations in the short term, and a transition to country-procured and financed products in the medium to long term. Leveraging the strengths of both systems, including forecasting and distribution functions, to improve the supply chain for NTD medicines and products is critical to sustainability. In countries where the NTD supply chain is integrated into the national supply chain, understanding the supply chain structures that facilitate or hamper sustainability, including how national systems incorporate new medicines, will be necessary.

## Materials and Methods

The Ascend programme is supporting national NTD programmes to develop and implement NTD supply chain capacity building plans in countries where the programme supports MDA. From April 2019 to March 2020, during the first year of the programme and prior to the onset of the COVID-19 pandemic, assessments took place in five countries: Chad, Ghana, Guinea Bissau, Liberia and Sierra Leone. These countries were selected based on various factors, including overall volume of NTD donated drugs to the country, representation of varying degrees of maturity in national NTD programmes and an ability to combine the field visits with a health system strengthening assessment.

An assessment tool was used to ensure consistent data collection and thematic analysis. The approach was aligned with the Dalberg Sustainability tool used for health system strengthening assessments. Focus areas (Table 1) were based on the technical expertise of the supply chain capacity building team and covered supply chain considerations for donated drugs found in the ITI Zithromax Guide for Programme Managers.^7^

**Table 1.** Focus areas for supply chain capacity assessment

**Assessment focus areas**
• Developing guidelines and standard operating procedures
• Owning procurement and supply systems
• Drug supply distribution network
• Staff and organisational support
• Planning and forecasting
• Data management and logistics management information system
• Inventory control procedures
• Warehousing and storage
• Quality assurance
• Transport and distribution (in-country)
• Reverse logistics
• Morbidity management and disease prevention
• Monitoring and evaluation and reporting
• Mass drug administration training

Assessments were led by the consortium's supply chain capacity building team with the support of country office staff and national NTD programme staff. Preceding field visits, an online review of the literature relevant to NTDs, as well as the national health system and the supply chain in each country for the last 3–5 years, was conducted. During field visits, semistructured key informant interviews (KIIs) were held with Ministry of Health and partner staff at national level and, where possible, with NTD stakeholders at health facilities (regional/provincial, district and subdistrict levels). KIIs focused on in-country procedures and on the last mile of the NTD supply chain. Health facilities were selected primarily based on accessibility within the timeframe for the field visit. Interviewees were selected based on their role and responsibility at the health facility and/or involvement with the NTD programme (e.g. on MDA campaigns). Health facilities have limited staff, and therefore many interviewees also support other health programmes. This consultative approach across different levels gave voice to the diverse stakeholders of the NTD supply chain. It gave those working at national level and delivering on the ground a chance to express challenges and opportunities.

Meetings were also held with representatives of other national health programmes, development partners and the national pharmaceutical board and/or School of Pharmacy (where available). These meetings helped the team to identify initiatives and opportunities in the wider health system and supply chain for cross learning and/or collaboration.

## Results

Assessments provided valuable information on where to build capacity and on how to address challenges in the NTD supply chain. The following sections of the paper summarise what was learnt in five of the focus areas across the countries assessed.

While the assessment primarily focused on PCT (preventive chemotherapy and transmission control)-NTD diseases, the results can be extrapolated to NTD case management diseases as the findings and recommendations relate to measures that address the strengthening of national systems. A key difference in the health supply chain for PC-NTD and case management treatments is the source of medicines. While PC drugs are supplied vertically through pharmaceutical donation programmes, medicines for case management can typically be found in the existing health system. Irrespective of supply sources and distribution modes, application of good supply chain management practices is required. An example is planning and forecasting, which requires granular data on treatment population and stock quantities.

### Planning and forecasting

Planning for MDA is a comprehensive process, of which drug quantification is a key element. Drug quantification needs to occur up to a year before drug treatments, in line with drug donation programmes and joint application processes. Typically, national census data plus the estimated annual percentage of population growth is used to assess the required number of treatments. This information is often outdated (by several years or even decades) and may not accurately account for changing population dynamics. For example, cross-border movement, internal migration of seasonal workers and cross-district travel by school-age children.

Last mile distribution planning occurs for each treatment round. One country highlighted an improvement in stock use by applying the previous year’s consumption data plus a buffer percentage to allocate stock.

### Drug supply distribution network, data management and logistics management information system

**Figure 1.**
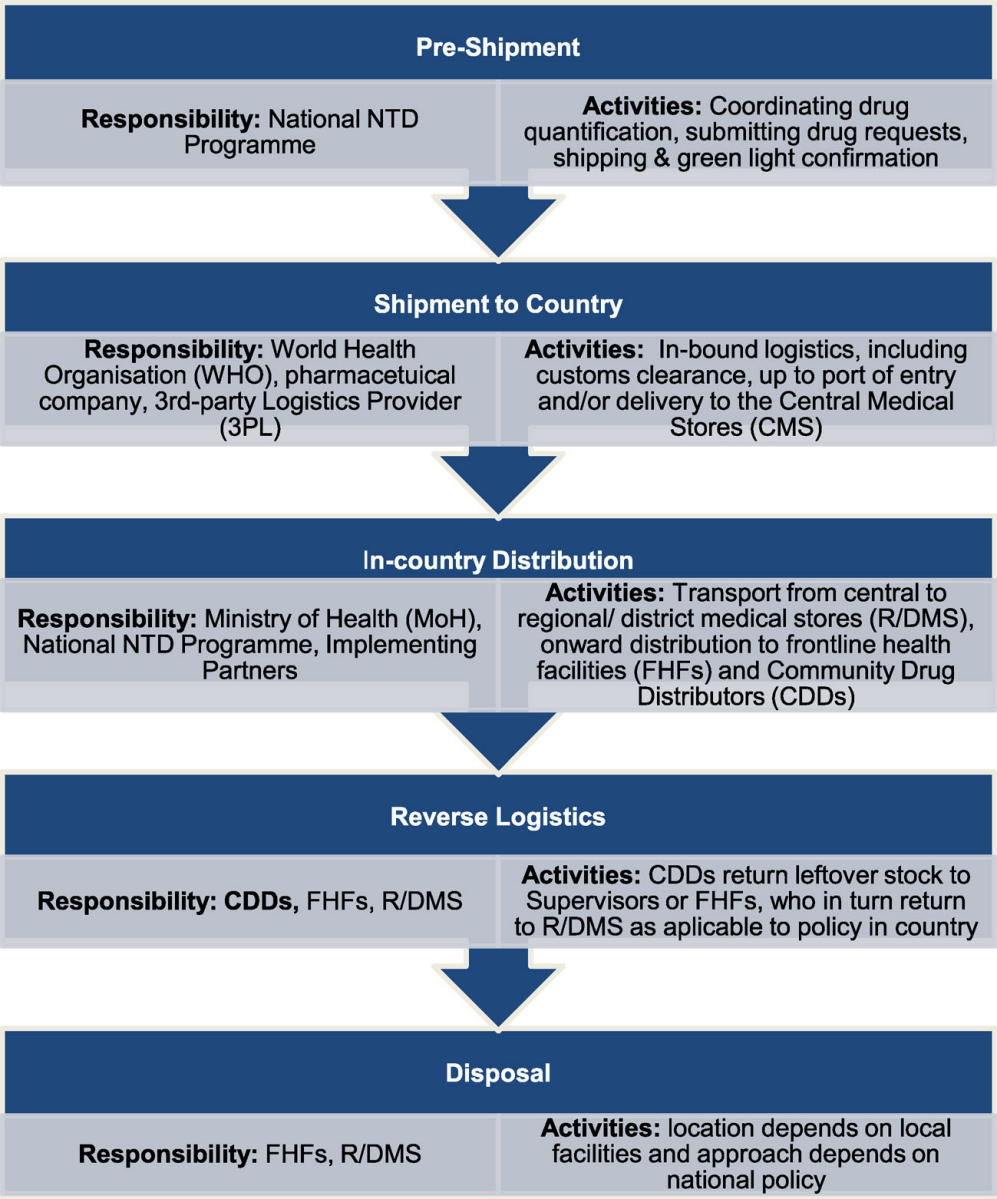
An example of an overview of the NTD supply chain for PCT donated drugs including key responsibilities and activities.

Distribution is a key step in the supply chain cycle, which starts preshipment and runs through to disposal. Figure 1 provides an overview of stages with key responsibilities and activities as an example. Data on leftover stock from previous treatment rounds are not easily retrievable, particularly from lower levels of the health system. Typically, there is an absence of an electronic logistics management information system (eLMIS) and MDA treatment records are kept by paper copies. The current verification practices of national NTD programmes are often resource-intensive, yet data on leftover stock are a requirement for annual drug applications.

At the time of assessment, all the countries assessed lacked integrated and reliable systems for stock management, tracking and reporting. However, countries are progressing at various stages with implementing (or expanding) eLMIS’. Reasons for lack of, or slow, progress can be financial as well as practical. For example, these systems require technology assets and support, reliable data access and human resource training.

The assessment found several scenarios. Some countries have an electronic system in place at the central medical stores (CMS) yet not at subnational level; national and subnational systems are different and not integrated; data systems and tools used by the NTD programme are operated separately to any national health and logistics information systems; and/or the process is paper-based.

Last mile drug distribution may be carried out by the national NTD programme. For example, drugs may be distributed to NTD supervisors through cascade training before MDA, or the NTD focal person may collect drugs from the medical stores. In one country it was found that the LMIS could not account for this process. The NTD focal person is not considered a ‘goods recipient’ in the system and as a result the system shows the drugs as ‘in stock’ after collection. In another country assessed, the LMIS consists of a set of paper-based forms and Microsoft Excel templates (Microsoft Corporation, Redmond, Washington, United States) do not integrate with any systems at the medical stores.

Data quality impacts the ability of national NTD programmes to plan and forecast, submit drug applications and allocate drug stocks, as well as learn from previous treatment rounds. This is relevant to all countries assessed irrelevant of maturity and/or size, although issues are heightened by paper-based systems and/or vast geographical coverage.

### Reverse logistics (and accountability for leftover stock)

Countries in receipt of donated drugs are required to keep track of and report on stock in their joint application package submission for the following year’s request for drugs. Therefore, retrieval of leftover stock following MDA (referred to as reverse logistics) is critical for NTD programmes.

NTD programmes do not have sustainable solutions for reverse logistics. Lack of equipment and poor infrastructure are known challenges for all countries assessed. The assessment also found that reverse logistics can rely on NTD staff at all levels, from NTD supervisors retrieving leftover stock from community-directed distributors to NTD national staff carrying out stock counts post-MDA. In one country, the NTD programme expressed concern that the time and costs of monitoring activities by national staff was a suboptimal practice.

Reverse logistics is not unique to NTD donated drugs, rather it is a common requirement for all health commodities. Measures to improve compliance with procedures and/or reduce the burden of monitoring need to be further explored. Opportunities can be explored with the national health supply system and/or other health programmes for (improved) coordination of these activities.

### Staff and organisational support

Assessments highlighted that NTD programmes have differing levels of support for supply chain management at the national level. For most national NTD programmes, the supply chain responsibilities are divided among NTD team members, rather than allocated as a separate role to a trained person. Some NTD programmes have a pharmacist within the national team. In all countries assessed, staff training for supply chain management remains a challenge regardless of the team structure.

A key recommendation for NTD programmes, which benefit from an NTD pharmacist within the team, is to raise the profile of NTDs within the national health system by raising the profile of the NTD pharmacist across the ministry, including the procurement and supply chain unit and the pharmacy board. This can be achieved through the NTD pharmacist taking an active role at the national level, to represent the NTD programme and facilitate integration of the programme into national systems such as its management information systems (LMIS for logistics and Health Management Information System (HMIS) for health). ​

## Discussion

As a result of the assessment findings and recommendations, the Ascend team contributes at the global level through partnerships such as the NTD Supply Chain Forum and the technical working group led by Expanded Special Project for Elimination of Neglected Tropical Diseases (ESPEN) Act to End NTD programme. This has provided opportunities to pool technical resources and avoid duplication of efforts, for example, on developing standard operating procedures (SOPs) for NTD supply chain management. The Ascend progamme is supporting several countries to review and provide feedback on the ESPEN SOPs, to adapt and adopt them to be country-specific and enhance the established MDA cascade training approach.

While there may be a desire to have one solution that can be easily replicated across all countries, there is no one-size-fits-all approach. Recommendations need to be contextualised for each country and aligned with health system-strengthening strategies. Ownership is with NTD programme managers, who may look to their counterparts in other health programmes and across the ministry, as well as to partners, to support implementation. Examples from the assessments can be found in Table 2.

**Table 2.** Example recommendations from the assessments

**Country**	**Focus area**	**Recommendation**	**Key benefit**	**Key dependencies**	**Implementation action**	**Risk to the programme (of no or incomplete action)**
Ghana; Liberia	Data management and LMIS	Configuration of LMIS to manage requirements for NTDs and staff training as required for use of system –including understanding and communication of the features/benefits available to NTDs	Data management and visibility	Management willingness, leadership and accountability to coordinate with technical team and follow up action to completion	National NTD programme (nominated team member) to coordinate with LMIS technical team (relevant team member)	Inhibits programme sustainability - logistics
Chad	Planning and forecasting	Provide feedback to ESPEN and review forecasting and reporting processes	Improved forecasting accuracy and reporting on NTD drugs	Collaboration between ESPEN, WHO and the NTD team	Review current forecasting and reporting processes	Inaccurate forecasting and reporting on NTD drug usage
Sierra Leone	Reverse logistics	Collaborate with NMSA to understand operations for reverse logistics	Leverage skills and support of NMSA; integration into national health supply chain system	Stakeholder willingness to drive collaboration/meetings and follow through on agreed actions	Stakeholder meetings to share current practices and develop options going forward	Reverse logistics is not carried out consistently or at all
Liberia	Staff and organisational support	Health commodity supply chain management training and skills development (national and subnational levels)	Improved drug handling throughout the supply chain	Management willingness and staff availability	Review/update MDA training content for drug supply chain management in line with SOPs; explore opportunities to collaborate with relevant institutions (e.g. WHO, School of Pharmacy)	Drug supply chain management is not fully understood at all levels and the quality and efficacy of drugs are at risk
		Build on existing SCM training delivered to pharmacists	Higher level degree of SCM skills among future pharmacists and direct contribution to strengthening the national health system	Collaboration among multiple parties	Contribute to updating the SCM component of the School of Pharmacy curriculum	Lack of SCM skills among future pharmacists directly impacting the NTD and national supply chain

Abbreviations: ESPEN, expanded special project for elimination of neglected tropical diseases; LMIS, logistics management information system; MDA, mass drug administration; NMSA, national medical supplies agency; NTD, neglected tropical disease; SCM, supply chain management; SOPs, standard operating procedures.

### Conclusion

The supply chain assessments supported through the Ascend West and Central Africa programme have highlighted key lessons in the last mile of the NTD supply chain. There is an opportunity for NTD programmes and national health supply chain teams to better understand how each other works and reinforce the role of qualified pharmacists and pharmacists’ technicians in the NTD supply chain. Integration of the NTD supply chain into national health systems should be an explicit objective of the NTD master plan at country level. An area that can be improved first is SOPs. This focus is shared at the global level through NTD partnerships: the NTD Supply Chain Forum and technical working groups of ESPEN and United States Agency for International Development Act to End NTD programme. Other opportunities include enhancing the established MDA cascade training as well as developing specific continuing professional development training for health workers.

Supply chain is recognised as a key element in the WHO ‘building blocks’ for health systems. The ongoing COVID-19 pandemic highlights the significant pressure faced by ministries of health. Building capacity in the last mile of the NTD supply chain means including hard to reach communities, which positively impacts strengthening of the national health system.

## Data Availability

None.
